# Ureteral Recurrence of the Endometrial Cancer: A Case Report

**DOI:** 10.1155/carm/5519713

**Published:** 2025-12-15

**Authors:** Hideo Yuki, Megumi Yokoyama, Takehiko Yamaguchi, Satoru Yamaguchi, Takao Kamai

**Affiliations:** ^1^ Department of Urology, Dokkyo Medical University Nikko Medical Center, 145-1 Moritomo, Nikko, Tochigi, 321-1298, Japan; ^2^ Department of Pathology, Dokkyo Medical University Nikko Medical Center, 145-1 Moritomo, Nikko, Tochigi, 321-1298, Japan; ^3^ Department of Surgery, Dokkyo Medical University Nikko Medical Center, 145-1 Moritomo, Nikko, Tochigi, 321-1298, Japan; ^4^ Department of Urology, Dokkyo Medical University, 880 Kitakobayashi Mibu-machi, Shimotsuga-gun, Tochigi, 321-0293, Japan, dokkyomed.ac.jp

## Abstract

Urinary tract complications can occasionally occur due to iatrogenic causes, particularly following surgical interventions for gynecological diseases. However, non‐iatrogenic ureteral injuries can also occur, for example, by the cancer invasion. This study reports the case of an 83‐year‐old woman who was referred to our hospital for right ureteral tumor. The patient’s chief complaint was macroscopic hematuria, and earlier, she had been referred to an urologist by a general physician. Right hydronephrosis and a lower ureteral tumor were observed on computed tomography and cystoscopy. Transurethral resection of the right ureteral orifice tumor had been carried out previously by an urologist. The condition was classified as urothelial cancer, Grade 2, pT1. A retroperitoneoscopic right nephroureterectomy was performed at our hospital. Extensive adhesion of the lower ureter to the surrounding tissue was detected. Consequently, the ureter, bladder cuff, and small intestine were resected *en bloc*. The pathological diagnosis was endometrial cancer recurrence. This is the first reported case of ureteral recurrence of the endometrial cancer. The recurrence or metastasis of endometrial cancer to the urinary tract is rare. Ureteral injuries are generally subtle; therefore, clinicians must maintain a high level of suspicion. Unrecognized or mishandled ureteral injuries might result in substantial complications. Herein, we present this rare case with some previously reported similar cases in the literature.

## 1. Introduction

Urinary tract complications can occasionally arise following gynecological treatment and are related to ureteral injuries and stenosis after uterine resection and bleeding cystitis after radiation. Urological issues may develop following treatment for malignant disorders, such as cervical cancer. We present a rare case of ureteral recurrence of endometrial cancer along with some previously reported cases of similar type.

## 2. Case Presentation

### 2.1. Patient Characteristics

**Figure 2:**
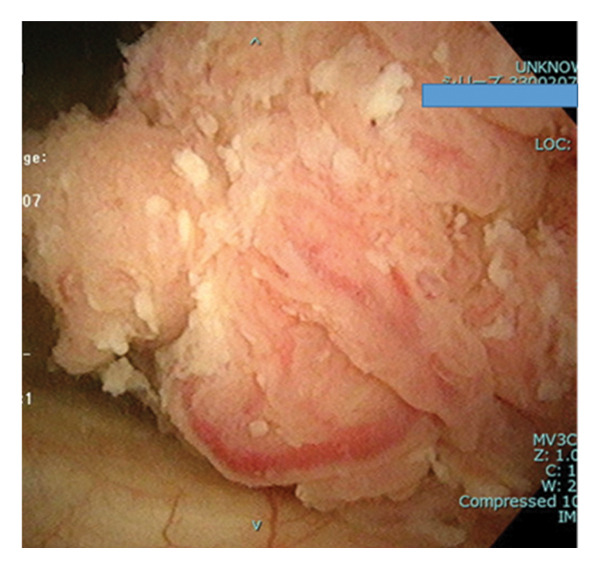
Nonpapillary bladder tumor confirmed by cystoscopy.

Our patient was an 83‐year‐old woman initially evaluated for macroscopic hematuria. She was previously treated for endometrial cancer, for which she was administered chemotherapy, and she underwent total hysterectomy 16 years prior. Prior to her current visit to our hospital, she had visited an urologist and was diagnosed with right lower ureteric malignancy (Figures 1(a), 1(b), and 2). Computed tomography revealed no additional tumors in the upper urinary system (Figure 1(c)). No cytological investigation of urine was performed. Transurethral excision of the bladder tumor was performed, and histology revealed urothelial cancer (UC), Grade 2, pT1. The patient was referred to our facility for treatment of residual ureteral carcinoma.

Figure 1Computed tomography (CT) scans of tumors. Enhanced CT scan showing (a) the bladder tumor (arrow), (b) the lower ureteral tumor (arrow), and (c) the right kidney.

**Figure:**
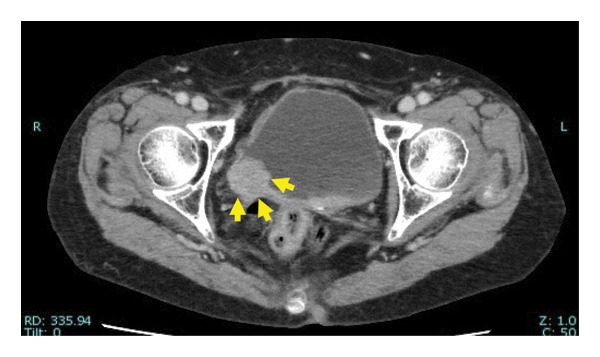
(a)

**Figure:**
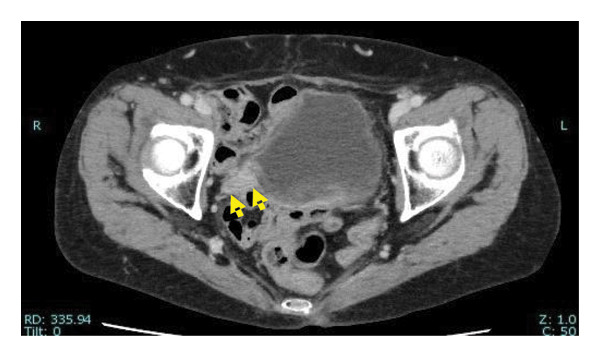
(b)

**Figure:**
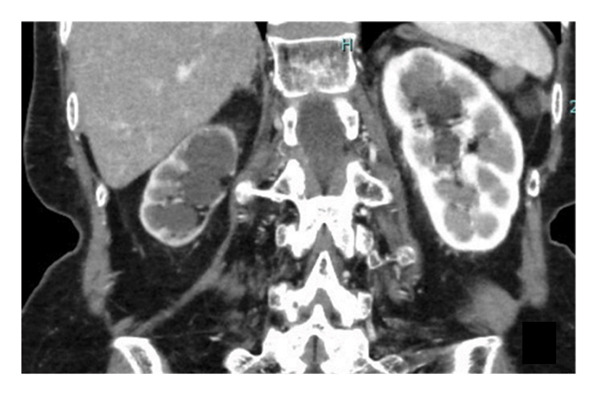
(c)

### 2.2. Surgical Technique

A retroperitoneoscopic radical nephroureterectomy was performed. During surgery, there were no complications in the region surrounding the kidney. Subsequently, a midline lower abdominal incision was made to resect the lower part of the ureter. Severe adhesion to surrounding tissues was observed around the ureter. Thus, the lower ureter, bladder cuff, and small intestine were resected *en bloc*. The total blood loss was 300 mL. On postoperative day (POD) 5, peroral intake was restarted. Urethral catheter was removed after cystography on POD 6. On POD 12, the peritoneal drainage tube was removed, and the patient was discharged on POD 23 (the extended hospital stay was due to the patient’s own social problems, not medical problems).

Hematoxylin and eosin (H&E) staining revealed luminal formation and cribriform tissue (Figure 3). Immunostaining revealed paired box (PAX) 8 positive (Figure 4(a)), GATA3 negative (Figure 4(b)), uroplakin II negative (Figure 4(c)), estrogen receptor positive (Figure 4(d)), and progesterone receptor positive (Figure 4(e)) results. Based on these findings, a pathological diagnosis of endometrial cancer was made, which differed from the diagnosis made at the previous hospital. Therefore, pathological rediagnosis was performed with tissues from the previous hospital. The results were similar to those obtained at our hospital (endometrial cancer and immunostaining). In addition, tumor dissemination to the renal pelvis and invasion of the small intestine were observed (Figure 5). For postoperative treatment, the patient was referred to a gynecological hospital.

Figure 3Hematoxylin & Eosin (H&E) staining. (a) H&E staining revealed luminal formation and cribriform tissue. (b) H&E staining (high magnification).

**Figure:**
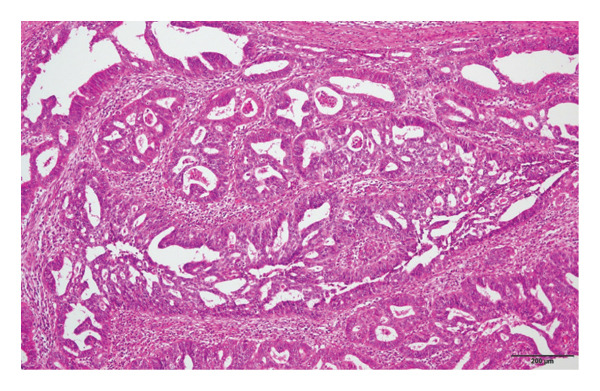
(a)

**Figure:**
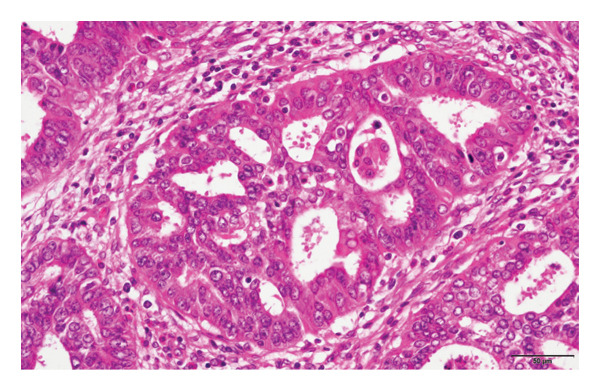
(b)

Figure 4Immunohistochemistry of the ureteral tumors. (a) Paired box (PAX) 8 positive. (b) GATA3 negative. (c) Uroplakin II negative. (d) Estrogen receptor positive. (e) Progesterone receptor positive.

**Figure:**
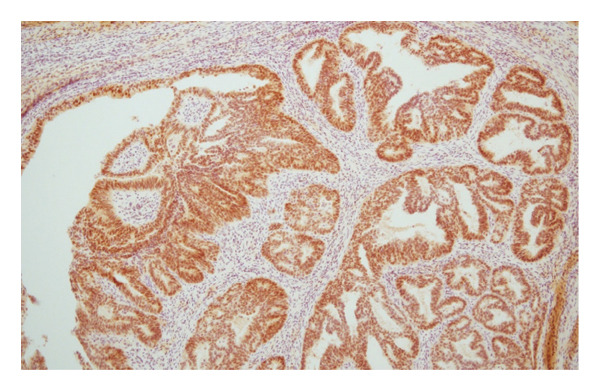
(a)

**Figure:**
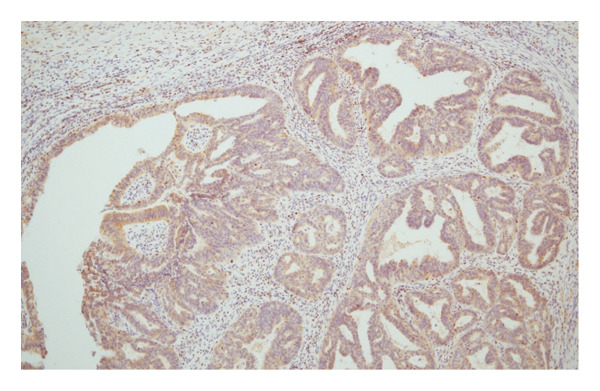
(b)

**Figure:**
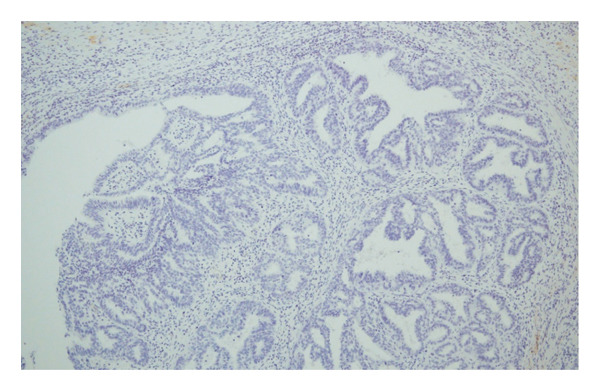
(c)

**Figure:**
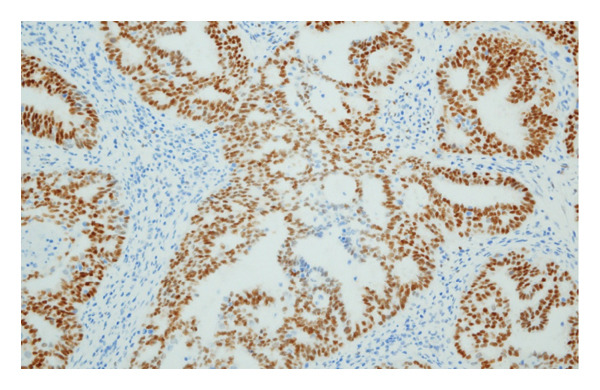
(d)

**Figure:**
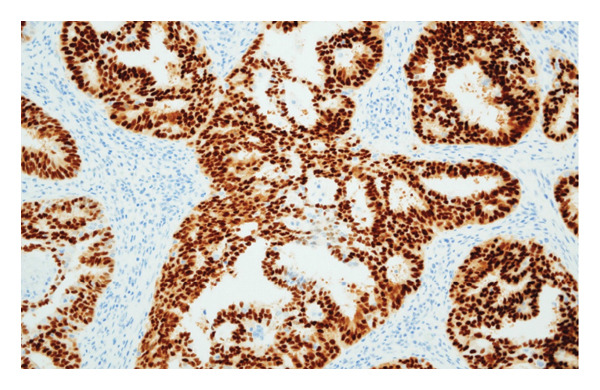
(e)

Figure 5Tumor invasion. (a) Tumor invasion of the small intestine was observed. (b) Tumor dissemination to the renal pelvis was observed (arrow).

**Figure:**
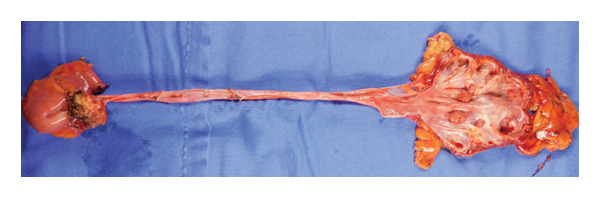
(a)

**Figure:**
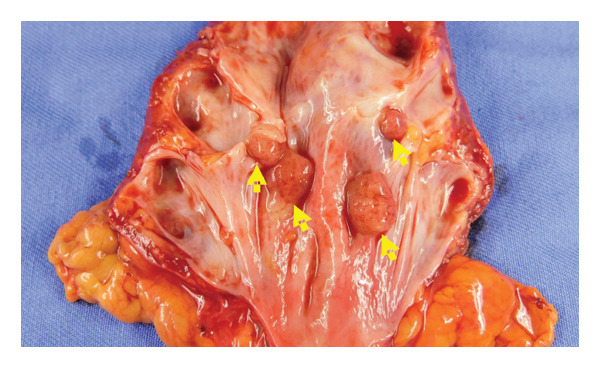
(b)

This study was conducted in accordance with the principles of the Declaration of Helsinki (1964) and approved by the hospital’s ethics committee. Written informed consent was obtained from the patient prior to surgery. Permission to publish this paper was obtained from the Director of the Dokkyo Medical University, Nikko Medical Center.

## 3. Discussion

The ureter is prone to complications from various diseases and medical procedures. For instance, an abdominal tumor can spread to the ureter, causing ureteral stricture, ureteral injury during abdominal surgeries, and ureteral stenosis following radiation therapy. Meanwhile, endometrial cancer exhibits diverse characteristics. Following treatment for endometrial cancer, only a small number of patients experience urinary tract problems. The age of onset of endometrial cancer is 40–50 years. The morbidity rate of endometrial cancer in Japan was 27.6/100,000 in 2019 [1]. The recurrence or metastasis of endometrial cancer to the urinary tract is rare [2–4]. Only four incidences of bladder recurrence or metastases have been recorded till date (Table 1) [5–8]. The present report is one of the first cases of ureteral recurrence. UC with luminal formation was diagnosed based on histological examination at another hospital, though immunohistochemistry was not performed. However, the histological examination at our hospital included immunostaining for endometrial cancer. The pathological diagnosis of these tissues is somewhat complicated. When complications occur in the urinary tract in patients treated for endometrial cancer, it is necessary to make clinical decisions, including careful pathological diagnoses, by an experienced pathologist specializing in urology. Moreover, depending on the patient’s medical history, it may be necessary to perform preoperative magnetic resonance imaging or positron emission tomography scans to examine the possibility of recurrence and/or metastasis of the tumor. In addition, it seems important to have an appropriate surgical plan and to reconfirm the pathological diagnosis before radical surgery.

**Table 1:** Cases of the recurrence or metastasis of endometrial cancer to the urinary tract.

No.	Age (years)	Stage	Surgical method for uterine cancer resection	Period until metastasis or recurrence (months)	Symptoms of recurrence	Treatment	Time course
1	68	1A	Total hysterectomy	16	Abnormal vaginal bleeding	Chemotherapy	Recurrence at the vaginal stump
2	49	4A	Radical hysterectomy, bilateral salpingo‐oophorectomy, pelvic lymph node dissection	Simultaneous	—	TURBT, endocrine therapy, radiation therapy	No recurrence for 10 months
3	72	1A	Radical hysterectomy, bilateral salpingo‐oophorectomy, pelvic lymph node dissection	50	Macroscopic hematuria	TURBT, chemotherapy	
4	71	1A	Semiradical hysterectomy, bilateral salpingo‐oophorectomy, pelvic lymph node dissection	72	—	TURBT, chemotherapy	Survived for 5 years despite postoperative recurrence
5 (this case)	83	2	Radical hysterectomy, bilateral salpingo‐oophorectomy, pelvic lymph node dissection	193	Macroscopic hematuria	TURBT, radical nephroureterectomy	No recurrence for 3 months

## 4. Conclusion

Here, we report a rare case of ureteral stricture due to ureteral recurrence of endometrial cancer. If complications occur in the ureter even after treatment for endometrial cancer, similar careful interventions need to be taken, as that after the treatment for cervical cancer.

## Disclosure

This study was conducted as part of the work of the authors at the Dokkyo Medical University Nikko Medical Center and Dokkyo Medical University.

## Conflicts of Interest

The authors declare no conflicts of interest.

## Funding

No funding was received for this manuscript.

## Data Availability

The data supporting the findings of this study are available from the corresponding author (H.Y.) upon reasonable request.
